# Supplementary material for: MELODI: Mining Enriched Literature Objects to Derive Intermediates
Source: Int J Epidemiol. 2018 Jan 12;47(2):369–79. doi: 10.1093/ije/dyx251 (PMC5913624; doi:10.1093/ije/dyx251)
Supplement: Supplementary Data [file dyx251_ije-2017-05-0596-file002.pdf]

# MELODI - Mining Enriched Literature Objects to Derive Intermediates

## Supplementary Material

### Supplementary table 1

High frequency (>150,000) SemMedDB terms that were removed as possible overlapping elements.

```
match (s:SDB_item) where s.i_freq > 150000 return s.name,s.i_freq order by
s.i_freq desc;
+-----------------------------------------------------------+
| s.name                                       | s.i_freq    |
+-----------------------------------------------------------+
| "Patients"                                   | 1.1766385E7 |
| "Therapeutic procedure"                      | 2890826.0   |
| "Cells"                                      | 1756153.0   |
| "Rattus norvegicus"                          | 1490929.0   |
| "Human"                                      | 1417176.0   |
| "Child"                                      | 1409253.0   |
| "Disease"                                    | 1152269.0   |
| "Woman"                                      | 1020950.0   |
| "Mus"                                        | 788379.0    |
| "Pharmaceutical Preparations"                | 681097.0    |
| "Brain"                                      | 674741.0    |
| "Neoplasm"                                   | 671717.0    |
| "Malignant Neoplasms"                        | 641918.0    |
| "Operative Surgical Procedures"              | 603799.0    |
| "Liver"                                      | 540926.0    |
| "Individual"                                 | 489076.0    |
| "Communicable Diseases"                      | 483483.0    |
| "Adult"                                      | 442605.0    |
| "Lung"                                       | 424022.0    |
| "Persons"                                    | 417370.0    |
| "Proteins"                                   | 403337.0    |
| "Lesion"                                     | 397681.0    |
| "Clinical Research"                          | 394102.0    |
| "Symptoms"                                   | 390574.0    |
| "Apoptosis"                                  | 381694.0    |
| "Serum"                                      | 353554.0    |
| "Body tissue"                                | 342993.0    |
| "Neurons"                                    | 336002.0    |
| "Male population group"                      | 333894.0    |
| "Genes"                                      | 320961.0    |
| "Injury"                                     | 312444.0    |
| "House mice"                                 | 289505.0    |
| "Infant"                                     | 279429.0    |
```

| | |
|---|---|
| "DNA" | 269543.0 |
| "Canis familiaris" | 267520.0 |
| "Excision" | 267145.0 |
| "Blood" | 265194.0 |
| "Obesity" | 264538.0 |
| "Antibodies" | 260010.0 |
| "Growth" | 256202.0 |
| "Muscle" | 252382.0 |
| "Kidney" | 245421.0 |
| "Malignant neoplasm of breast" | 241219.0 |
| "Animals" | 240389.0 |
| "Eye" | 236654.0 |
| "Heart" | 234823.0 |
| "Cell Line" | 234750.0 |
| "Complication" | 234074.0 |
| "response" | 232681.0 |
| "Injection procedure" | 231834.0 |
| "Pharmacotherapy" | 222967.0 |
| "Study models" | 218609.0 |
| "cytokine" | 217142.0 |
| "Hypertensive disease" | 214464.0 |
| "Adolescent" | 211644.0 |
| "Oryctolagus cuniculus" | 211396.0 |
| "Functional disorder" | 202367.0 |
| "Asthma" | 198127.0 |
| "Plasma" | 196611.0 |
| "Analysis" | 195707.0 |
| "Blood Vessels" | 193426.0 |
| "Syndrome" | 193414.0 |
| "Expression procedure" | 191459.0 |
| "Inflammation" | 190337.0 |
| "Family" | 189865.0 |
| "Enzymes" | 189542.0 |
| "Elderly" | 188966.0 |
| "Assay" | 183594.0 |
| "Diabetes" | 181387.0 |
| "Mothers" | 181207.0 |
| "Infection" | 181066.0 |
| "Detection" | 180400.0 |
| "Cattle" | 175424.0 |
| "Glucose" | 172343.0 |
| "Infant, Newborn" | 172193.0 |
| "Water" | 167885.0 |
| "Antigens" | 167731.0 |
| "Escherichia coli" | 166711.0 |
| "Diabetes Mellitus, Non-Insulin-Dependent" | 166429.0 |
| "Mutation" | 164359.0 |
| "Ethanol" | 162985.0 |
| "Rheumatoid Arthritis" | 160301.0 |
| "Radiation therapy" | 160129.0 |
| "Obstruction" | 156260.0 |
| "Pain" | 155477.0 |
| "Family suidae" | 155318.0 |

```
| "receptor"                                              | 154339.0    |
| "Placebos"                                              | 152987.0    |
+----------------------------------------------------------------------+
88 rows
```

## Supplementary table 2

Comparison of MELODI and similar tools

| | **MELODI** | **Arrowsmith** [1] | **SemBT (Closed Discovery)** [2] |
|---|---|---|---|
| Article limit | 1,000,000 | 25,000 | N/A |
| Source of text | PubMed | PubMed | SemMedDB |
| Source of text objects | SemMedDB and MeSH (2016) | Custom generated from MEDLINE (2005) [3] | SemMedDB |
| Types of text | Titles, abstracts and MeSH terms | Titles | Titles and abstracts |
| Enrichment method | Corrected P-value of local vs global frequencies | Custom formula [3] | Unknown |
| Data storage | Neo4j and MySQL | N/A | MySQL |
| User account | Yes | No | No |
| Export results | Yes | No | No |
| Share results page | Yes | No | No |

Supplementary Figure 1 – A three article ‘article set’

Each article set contains relationships to a set number of articles (green nodes) each of which has pre-defined relationships with MeSH/SemMedDB objects. Purple nodes represent MeSH terms, red and yellow the SemMedDB triples and concepts respectively

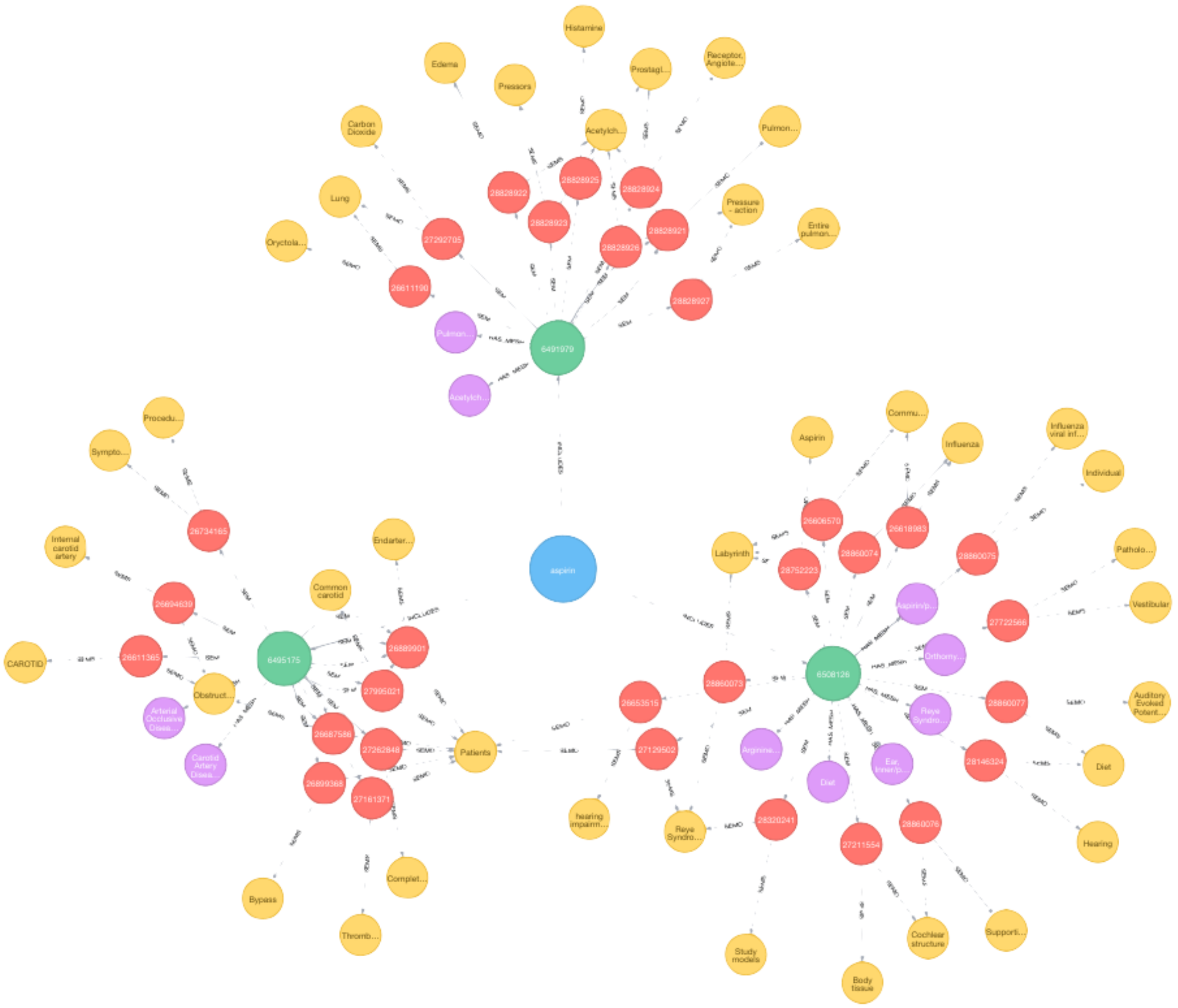

Supplementary Figure 2 - Data flow within MELODI

Tasks are created each time an article set is formed or two article sets are compared. These tasks are passed to the task manager redis (ref) which then communicates with Celery, the asynchronous job queue. If available, a worker is assigned to the task which on completion updates the databases. If no worker is available, the task is held in a queue. Tasks continuously update the MySQL database to provide real-time job status updates.

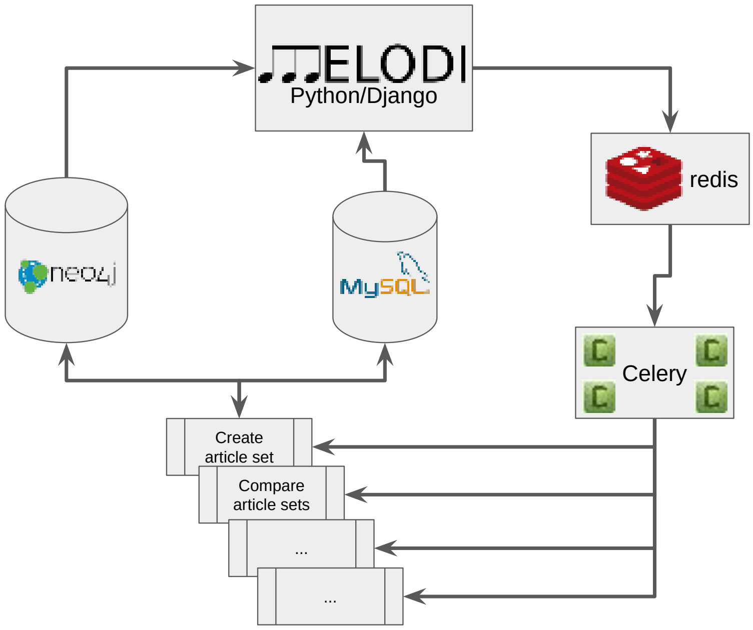

Supplementary Figure 3 - An example of an overlapping enriched object

In this example two article sets were compared, one focused around ‘Milk’ and the other ‘Prostate Cancer’. The term IGF-1 is part of an enriched SemMedDB triple in each of the article sets. It is identified as overlapping as it is the object in the triple for article set A and the subject in the triple for article set B.

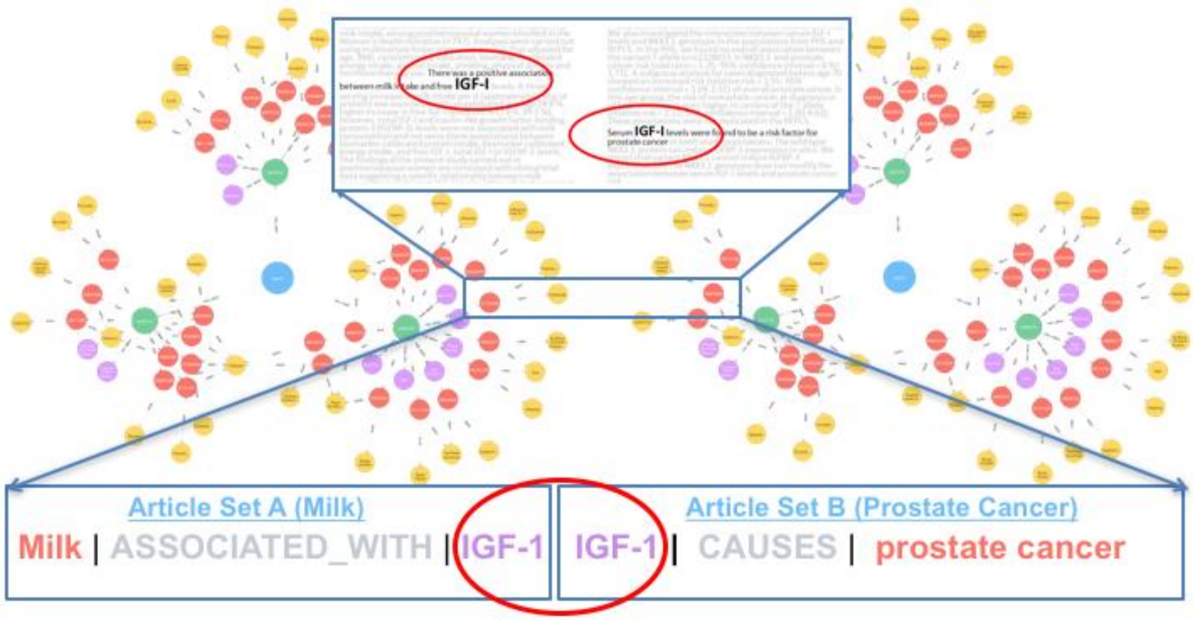

Supplementary Figure 4 - An example of filtering results

Results are filtered both automatically and via user input. Both are necessary to reduce often large numbers of enriched overlapping concepts to more manageable numbers. The automatic filtering step is based purely on the number of overlaps, a high number increasing the stringency of the filter thresholds and a low number decreasing it. User filtering is achieved by either positively or negatively filtering the results by concepts and predicates. In this case seven overlapping concepts were removed from the results.

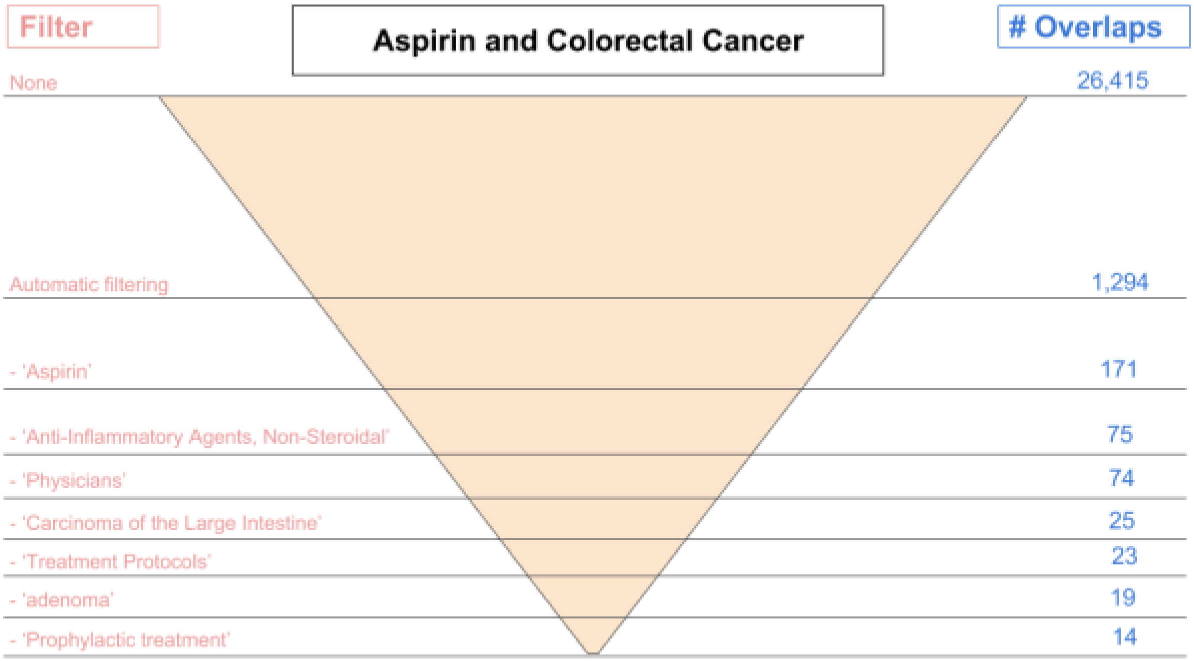

# References


1. Smalheiser, N. R., Torvik, V. I. & Zhou, W. Arrowsmith two-node search interface: a tutorial on finding meaningful links between two disparate sets of articles in MEDLINE. *Comput. Methods Programs Biomed.* **94,** 190–197 (2009).
2. Hristovski, D., Dinevski, D., Kastrin, A. & Rindflesch, T. C. Biomedical question answering using semantic relations. *BMC Bioinformatics* **16,** 6 (2015).
3. Torvik, V. I. & Smalheiser, N. R. A quantitative model for linking two disparate sets of articles in MEDLINE. *Bioinformatics* **23,** 1658–1665 (2007).
